# Supplementary material for: More replenishment than priming loss of soil organic carbon with additional carbon input
Source: Nat Commun. 2018 Aug 9;9:3175. doi: 10.1038/s41467-018-05667-7 (PMC6085371; doi:10.1038/s41467-018-05667-7)
Supplement: Supplementary file 1 — Supplementary Information [file 41467_2018_5667_MOESM1_ESM.pdf]

More Replenishment than Priming Loss of Soil Organic Carbon with Additional Carbon Input

Liang et al.

Supplementary Information includes:

Supplementary Figs. 1 – 9

Supplementary Data 1 – 6 (in separate Excel files)

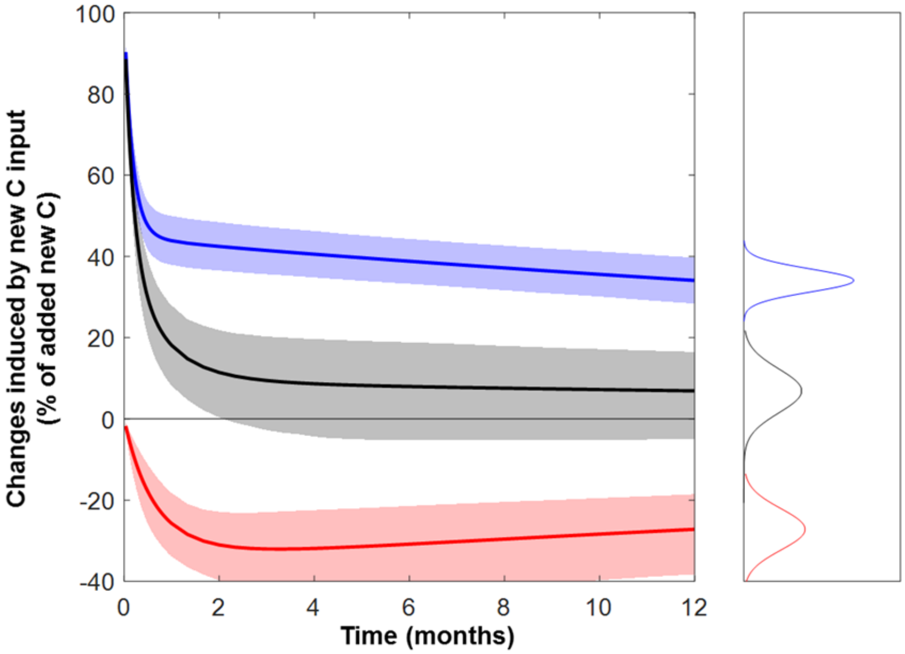

Supplementary Figure 1 | Time courses of effects of replenishment (blue) and priming (red) on SOC, and the net SOC change (black) with a one-time new C input at the beginning. Lines are mean values of model simulations and shading areas are the ranges from 2.5th to 97.5th percentiles (i.e., 95% range). The box on the right shows the distributions of cumulative changes at the end of the first year (i.e., annual changes). The simulations are from the same example as shown in **Fig. 3**.

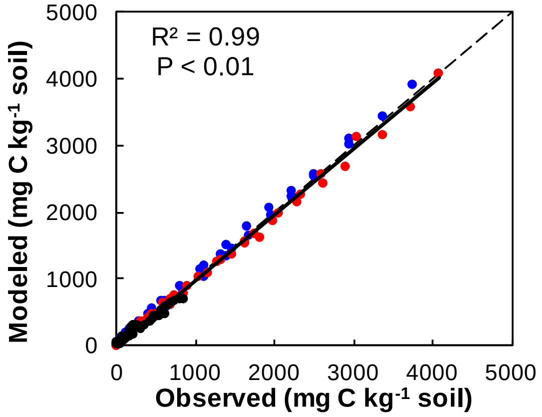

Supplementary Figure 2 | The first out-of-sample validation of the interactive model. The results show that the calibrated model by data of one particular new C addition amount can reproduce the results of other new C addition amount. y-axis shows mean values of simulated CO_2 emissions for studies in data group II by the interactive model optimized against the corresponding studies in data group I. Blue, CO_2 emission from old SOC at control; Red, CO_2 emission from old SOC at new C addition treatment; Black, CO_2 emission from added new C. Dashed line: 1:1 line; Solid line: linear regression line.

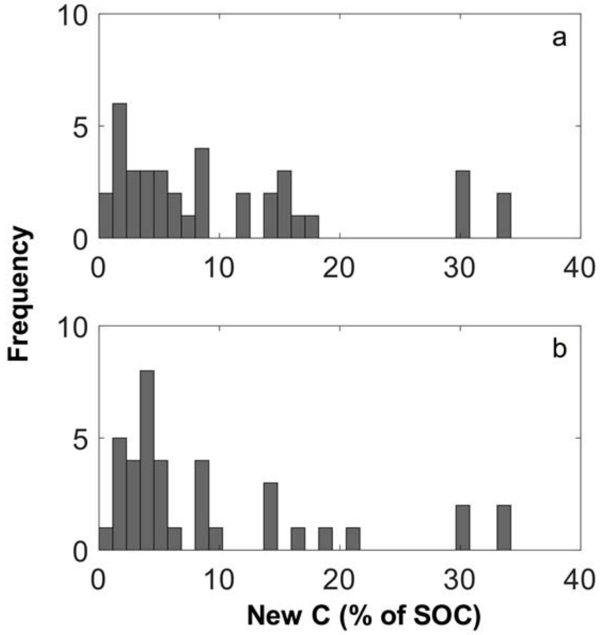

Supplementary Figure 3 | The distribution of the added new C amount in the two groups for the second method for validation. a is for the training and **b** is for the validation, and the two groups have similar distributions.

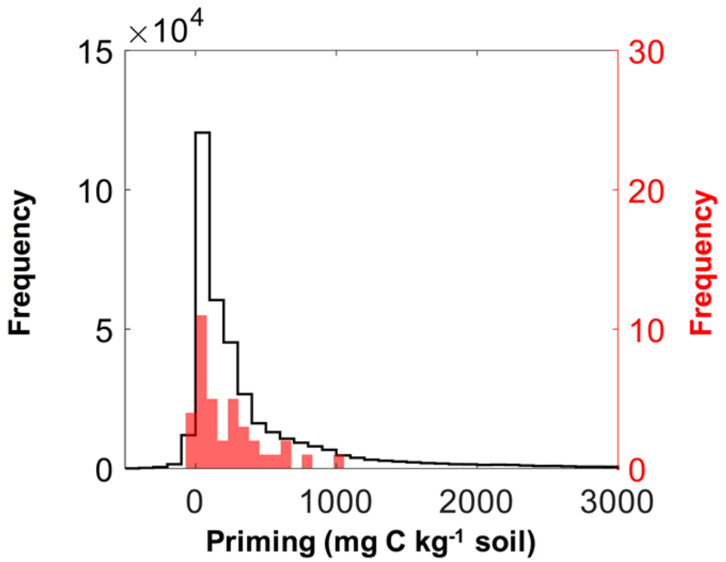

Supplementary Figure 4 | The second method for validation. The x-axis is the cumulative priming effect at the end of experiments. It is shown that the model predictions and observations had very similar distributions, validating that the calibrated interactive model has a high ability to simulate SOC decomposition with priming. The black stairs and the red bars are modeled and observed, respectively.

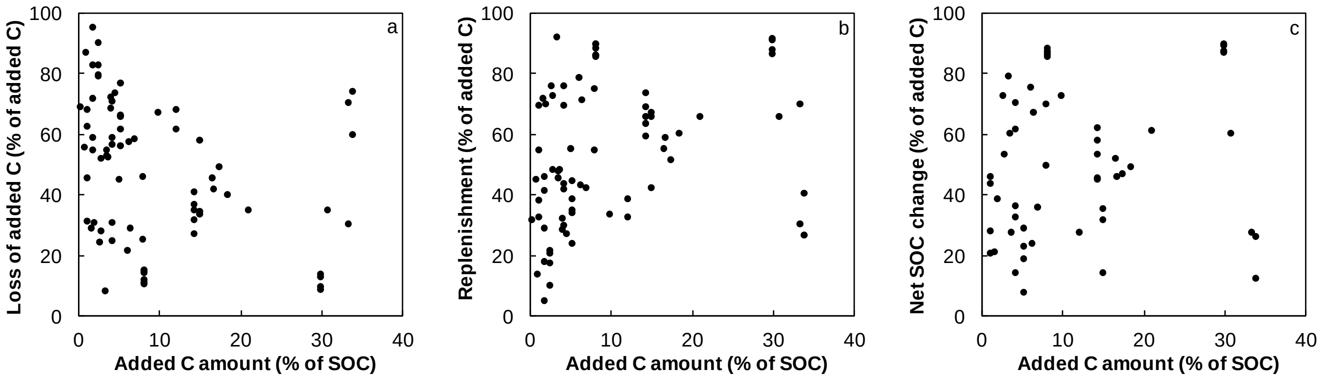

Supplementary Figure 5 | Dependence of C fluxes on the amount of added substrate-C. a, loss of added C; **b,** replenishment; **c,** net SOC change. It is shown that the results are not dependent on the added C amount.

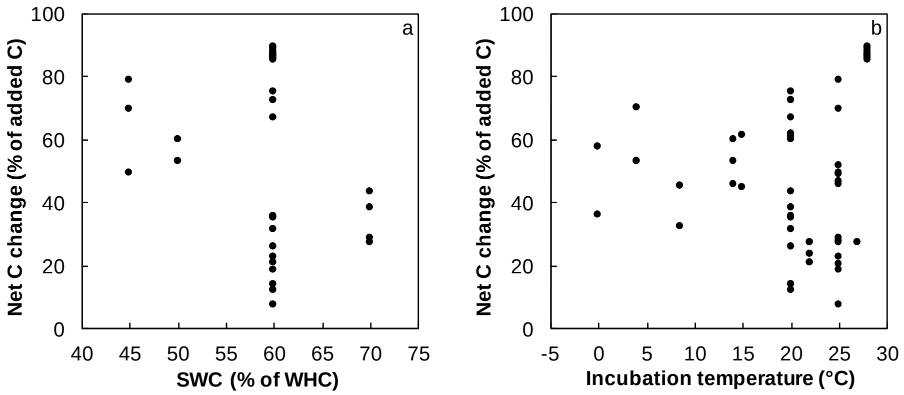

Supplementary Figure 6 | Dependence of the net SOC change on soil water content (SWC, % of water holding capacity (WHC)) and incubation temperature. It is shown that the net SOC change by new C addition is not dependent on incubation SWC or temperature.

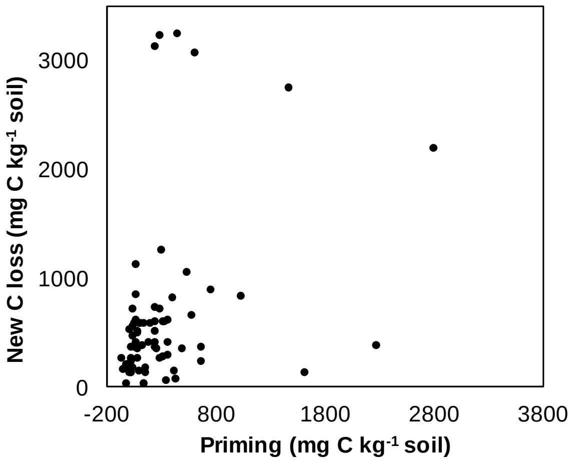

Supplementary Figure 7 | The cumulative C loss of the new C against priming at the end of experiment in all the collected datasets.

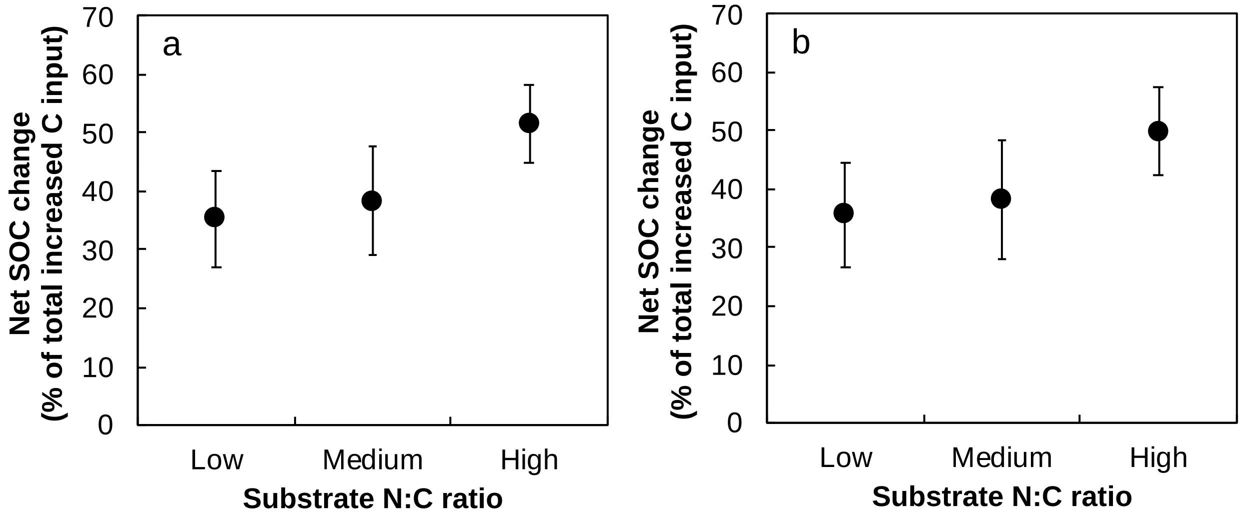

Supplementary Figure 8 | Dependence of the net SOC change on substrate N:C ratio after one-year continuously increased C inputs. a and **b** show the results with step and gradual increase in C inputs, respectively. The low, medium and high N:C ratios are corresponding to the groups in Figure 5. Mean ± 95% confidence interval.

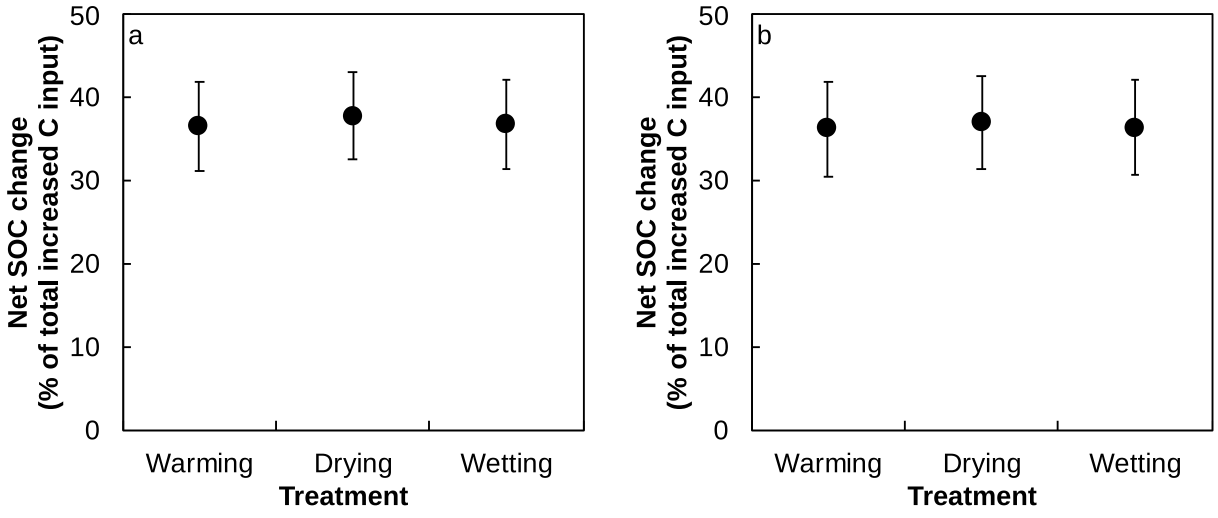

Supplementary Figure 9 | Modeling experiment showing the net SOC change by increased C inputs with temperature and moisture fluctuations. a, step increase in C input; **b,** gradual increase in C input. Mean ± 95% confidence interval.
