# Supplementary material for: More replenishment than priming loss of soil organic carbon with additional carbon input
Source: Nat Commun. 2018 Aug 9;9:3175. doi: 10.1038/s41467-018-05667-7 (PMC6085371; doi:10.1038/s41467-018-05667-7)
Supplement: Supplementary file 3 — Description of Additional Supplementary Files [file 41467_2018_5667_MOESM3_ESM.pdf]

Description of Additional Supplementary Files

File Name: Supplementary Data 1
Description: Summary of the collected data. Ecosystem type, soil sample location, country, type of added substrate, soil organic carbon content, amount of added carbon and nitrogen-to-carbon ratio of added substrate are provided.

File Name: Supplementary Data 2
Description: Details of the collected data. Time series of CO_2 emissions from soil organic carbon and added substrate are provided.

File Name: Supplementary Data 3
Description: Maximum Likelihood Estimates (MLEs) of calibrated parameters of the conventional model.

File Name: Supplementary Data 4
Description: Maximum Likelihood Estimates (MLEs) of calibrated parameters of the interactive model.

File Name: Supplementary Data 5
Description: Maximum Likelihood Estimates (MLEs) of calibrated parameters of the regular Michaelis-Menten model.

File Name: Supplementary Data 6
Description: Maximum Likelihood Estimates (MLEs) of calibrated parameters of the reverse Michaelis-Menten model.
